# Anesthetic Management of a Child with Mitochondrial Neurogastrointestinal Encephalopathy

**DOI:** 10.1155/2015/453714

**Published:** 2015-06-01

**Authors:** Vianey Q. Casarez, Acsa M. Zavala, Pascal Owusu-Agyemang, Katherine Hagan

**Affiliations:** Department of Anesthesiology & Perioperative Medicine, The University of Texas MD Anderson Cancer Center, 1515 Holcombe Boulevard, Unit 409, Houston, TX 77030, USA

## Abstract

Mitochondrial neurogastrointestinal encephalomyopathy (MNGIE) is an autosomal recessive disorder associated with deficiency of thymidine phosphorylase (TP). Associated manifestations include visual and hearing impairments, peripheral neuropathies, leukoencephalopathy, and malnutrition from concomitant gastrointestinal dysmotility and pseudoobstruction. Given the altered metabolic state in these patients, specific consideration of medication selection is advised. This case report will describe the anesthetic management used in a 10-year-old girl with MNGIE. She had multiple anesthetics while undergoing allogeneic hematopoietic stem cell transplantation. This case report will discuss the successful repeated use of the same anesthetic in this pediatric patient, with the avoidance of volatile anesthetic agents, propofol, and muscle relaxant.

## 1. Introduction

MNGIE is a rare autosomal recessive genetic disorder directly associated with a deficiency of thymidine phosphorylase (TP) [1–4]. MNGIE results from a mutation in the TYMP gene (ECGF 1 gene) that encodes for thymidine phosphorylase (TP). This mutation results in a reduction or elimination of TP activity. TP breaks down thymidine and regulates the levels of thymidine and deoxyuridine in the body via negative feedback loop, so shortage of TP allows thymidine to build up [2–4]. Mitochondria use thymidine to build new molecules of mitochondrial DNA (mtDNA), so the excess of thymidine can result in mutations that damage the replication, maintenance, and repair of mtDNA. Patients with MNGIE have low levels of TP; therefore, toxic levels of thymidine and deoxyuridine are responsible for causing irreversible mitochondrial mutations in these patients [4]. These mutations can result in a decrease in ATP production via oxidative phosphorylation in the respiratory chain found in mitochondria, affecting tissues that have high energy demands including cardiac, nervous, and skeletal muscle tissue. Mutated mitochondrial protein production can also affect the tissues where the protein is found, resulting in GI and hepatic disease.

Clinically, this disorder is difficult to diagnose given the array of symptoms that present concurrently with one another. These patients typically present with visual and hearing impairments, peripheral neuropathy, and leukoencephalopathy. Other prominent symptoms include malnutrition from concomitant gastrointestinal dysmotility and pseudoobstruction [3–5]. Given the concerning gastrointestinal symptoms that ensue in these patients, MNGIE patients commonly require anesthetic services for exploratory laparotomies and diagnostic endoscopies throughout the progression of their disease.

MNGIE patients are now being offered an option to undergo allogeneic hematopoietic stem cell transplantation (HSCT) [2–4]. Although this innovation is new and has only been used successfully in nine MNGIE patients, this treatment is yielding promising results as it aims to normalize thymidine phosphorylase (TP) levels [2, 6]. Studies have shown that early HSCT treatment in these patients helps deter disease progression by reversing the toxic nucleoside levels of thymidine and deoxyuridine found in patients with this disorder [2–4]. While stem cell transplantation has not been found to reverse mitochondrial mutations or help with neurological symptoms, improved gastrointestinal relief has been noted with this treatment. For this reason, HSCT treatments have been found to decrease the morbidity and mortality that are attributed to the diminished gastrointestinal health found in MNGIE patients [2].

Given the altered metabolic state, medications administered to these patients should be carefully selected [7]. There is no preferred anesthetic for these patients. Although the medical literature describes many anesthetic approaches, individuals with MNGIE respond atypically to common anesthetic agents [8, 9]. With written permission from this patient's father and local IRB approval, this case report presents our approach in the care of a 10-year-old girl with MNGIE undergoing stem cell transplantation.

## 2. Case Presentation

A 10-year-old girl previously diagnosed with MNGIE disease presented to the anesthesia assessment center for preoperative evaluation the day before her scheduled surgery. The patient was scheduled to undergo anesthesia for placement of a central venous catheter for her upcoming stem cell transplantation. The patient presented with hearing deficits, visual abnormalities, cognitive delays, small stature, chronic malnutrition, and unsteady gait. Given her complicated disease process, the decision was made to admit the patient to the hospital that evening in anticipation for surgery the following morning. In an effort to maintain adequate hydration and oxygenation during the preoperative fast, an intravenous saline solution with 5% dextrose was administered as a continuous infusion overnight.

The patient weighed 17 kg and was 109 cm tall. Her previous surgical history included a right cochlear implant four years priorly and no anesthetic complications at that time. Preoperatively, the patient was awake, cachectic, and frail. She was accompanied by her father, sitting up in bed, and spontaneously breathing room air. Her vital signs included tachycardia of 124 beats per minute, her airway exam was normal, and her laboratory examination showed slight hypoalbuminemia of 3 g/dL and low total protein of 5.4 g/dL. The anesthesia plan was to intubate the patient as the patient had GI dysmotility and abdominal pain.

Upon arrival to the operating room suite, monitors were placed, preoxygenation was provided, and general anesthesia was induced. The induction agents of choice included 0.15 mg/kg of midazolam, 3 mcg/kg of fentanyl, and 1 mg/kg of ketamine. Continuous infusions of 0.7 mcg/kg/min of dexmedetomidine and 15 mcg/kg/min of ketamine were also initiated at the start of induction and maintained throughout the case. After adequate depth of anesthesia was established, the patient was easily intubated with a 5.0 mm cuffed endotracheal tube following topical laryngotracheal anesthesia with an LTA using 1 cc of 4% lidocaine. The use of volatile anesthetic agents, muscle relaxants, and propofol was omitted in the care of this patient. The patient remained hemodynamically stable throughout the procedure. Once a left subclavian triple lumen catheter was placed, the dexmedetomidine and ketamine infusions were both discontinued. Spontaneous ventilation was established; the patient was extubated shortly thereafter and transported to the postoperative recovery unit.

Throughout this patient's hospital stay, our anesthetic team had the privilege of caring for this patient on two other occasions and the same anesthetic plan was utilized. The other two procedures included a repeated central line venous insertion and a liver biopsy. The liver biopsy was performed after she developed abnormal liver function tests (increased AST, ALT, alkaline phosphate, and bilirubin) following her stem cell transplant from a matched unrelated donor. Due to the patient's young age and the anticipated discomfort of the procedure, the decision was made to anesthetize the patient for the liver biopsy. After each of these anesthetics, the patient was closely monitored and no apparent postoperative complications were reported.

## 3. Discussion

Patients with mitochondrial abnormalities create evident challenges for anesthetic providers for a multitude of reasons. Not only do mitochondrial syndromes involve multisystem organ impairments, but also the lack of well-defined guidelines in the care of these patients makes it even more cumbersome. There is minimal published information on the anesthetic management of patients with MNGIE; thus, anesthesia personnel are limited not only to making clinical decisions and medication selections based upon the patient's past medical history but also to limited published reports on anesthetic effects on mitochondria [8].

During the preoperative period, the patient was provided with continuous intravenous solution to prevent dehydration. A retrospective case review published in the British Journal of Anaesthesia found that preoperative fasting can be detrimental to patients with mitochondrial disease when not adequately hydrated during the fasting period. Recommendations in that study support the use of lactate-free intravenous fluids with dextrose to prevent lactic acidosis in all patients with mitochondrial disorders. The article further notes that excessive glucose oxidation can increase levels of lactate in the blood when normal glucose levels are not maintained [7, 10]. The normal glucose levels would be 70–110 fasting and less than 140 mg/dL two hours after eating a meal.

When comparing the most commonly used induction agents in anesthesia, propofol has been discovered to affect mitochondrial function the most when compared to ketamine, etomidate, and barbiturates [8, 11, 12]. While the latter three agents have been found to only inhibit one mechanism of mitochondrial function, propofol has been found to depress mitochondrial function via four different pathways [8]. Although some case reports have successfully reported the use of propofol in patients with mitochondrial disease, other studies have found an increased sensitivity of propofol infusion syndrome and delayed sequela from the use of propofol in mitochondrial patients [8, 11–13]. For this reason, we decided to avoid the use of propofol.

Volatile anesthetic gases have also been found to inhibit mitochondria [13]. Although anesthetic gases are minimally metabolized, avoiding the use of volatile anesthetics is ideal in patients of mitochondrial disease given the mitochondrial mutations found with these syndromes. Metabolic derangements alter the utilization of full oxygen capacity and ATP production and ultimately increase the sensitivity that these patients have to volatile anesthetics [8, 14]. The use of sevoflurane has been suggested as the maintenance anesthetic of choice for mitochondrial patients; however, sevoflurane sensitivity has also been linked to some patients with mitochondrial disease [13].

To date, there is no data suggesting that muscle relaxants or narcotics contribute to the inhibition of mitochondrial pathways [8]. Although neuromuscular blocking agents were not used in this patient, the decision was solely based on the type of procedures conducted and the proposed lengths of surgery. We were able to successfully secure an endotracheal tube with the combination of laryngotracheal anesthesia, midazolam, fentanyl, and ketamine in this patient. By utilizing current literature to develop the anesthetic plan, this case report effectively demonstrates the specific considerations that were taken to avoid further deterioration of this patient's ongoing metabolic derangements.

Adequate hydration, oxygenation, and limited stress are important in the preoperative, intraoperative, and postoperative care of these patients [7]. Choosing the safest combination of anesthetic agents for patients with MNGIE is challenging given that all general anesthetic medications have been found to inhibit mitochondrial function to varying degrees [8, 11, 12, 15]. Until more studies are conducted regarding the anesthetic management of patients with specific mitochondrial disorders, anesthetic providers are expected to devise an individualized anesthetic care plan that is safest for the patient based on current recommendations, current medical history, and their best clinical judgment.
